# Population Structure of* Leishmania tropica* Causing Anthroponotic Cutaneous Leishmaniasis in Southern Iran by PCR-RFLP of Kinetoplastid DNA

**DOI:** 10.1155/2018/6049198

**Published:** 2018-06-06

**Authors:** Mohammad Amin Ghatee, Hossein Mirhendi, Masoud Marashifard, Zahra Kanannejad, Walter R. Taylor, Iraj Sharifi

**Affiliations:** ^1^Cellular and Molecular Research Center, Yasuj University of Medical Sciences, Yasuj, Iran; ^2^Department of Parasitology, School of Medicine, Yasuj University of Medical Sciences, Yasuj, Iran; ^3^Department of Parasitology and Mycology, School of Medicine, Isfahan University of Medical Sciences, Isfahan, Iran; ^4^Department of Immunology, School of Medicine, Shiraz University of Medical Sciences, Shiraz, Iran; ^5^Mahidol Oxford Tropical Medicine Research Unit, Bangkok, Thailand; ^6^Centre for Tropical Medicine and Global Health, Nuffield Department of Medicine, University of Oxford, UK; ^7^Leishmaniasis Research Center, Kerman University of Medical Sciences, Kerman, Iran

## Abstract

Iran is one of the six countries with the most cutaneous leishmaniasis (CL) patients. Understanding better the genotypes of the parasite population in relation to geography and climate is critical to achieving better CL control. We aimed to characterise the population structure of* Leishmania tropica*, the cause of anthroponotic cutaneous leishmaniasis (ACL), from important foci in southeast (Bam and Kerman) and southwest (Shiraz) Iran. A total of 39* L. tropica* isolates from ACL patients from southeast (Bam 14, Kerman 12) and southwest (Shiraz 13) Iran were analysed by polymerase chain reaction restriction fragment length polymorphism (PCR-RFLP) of the kinetoplast DNA (kDNA) using restriction enzymes* Msp*I (*HpaII*) and* Cla*I. 37 genotypes were identified among south Iran* L. tropica* isolates. The unweighted pair group method with arithmetic mean (UPGMA) tree obtained from the banding patterns of* Cla*I digested kDNA RFLP distinguished southeast from and southwest* L. tropica *isolates with some subclustering but the* Msp*I derived tree showed greater discrimination with greater subclustering and divergence of the two foci of southeast region but with some overlapping. Although a monophyletic structure has been defined for southeast* L. tropica*, isolates from two foci of southeast Iran were partly discriminated in the current study.

## 1. Introduction

Leishmaniasis is a global vector-borne disease caused by the protozoa belonging to the* Leishmania* genus that is transmitted through the bite of the* phlebotomine* sand fly species [1]. Leishmaniasis is categorised by a spectrum of clinical manifestations from cutaneous and mucocutaneous to visceral forms. Cutaneous leishmaniasis (CL) is the most common form of the disease with 0.7 to 1.2 million new cases per year being reported worldwide [2]. CL causes skin lesions on the exposed sites of the body, leaving life-long scars, notable disability, and psychological distress and has important negative social and economic consequences. Although CL is considered as a significant public health challenge, it remains a neglected disease [3].

Over two-thirds of new CL cases occur in six countries: Afghanistan, Algeria, Brazil, Colombia, Syria, and Iran [4]. Two epidemiological forms of CL are present in Iran, anthroponotic (ACL) and zoonotic cutaneous leishmaniasis (ZCL), and are caused by* L. tropica* and* L. major*, respectively. ZCL extends mostly in rural regions of the northeast, the central, the west, and the southwest of the country and occurs in some parts of southern and southeast provinces as well [5–14].

**Figure 1:**
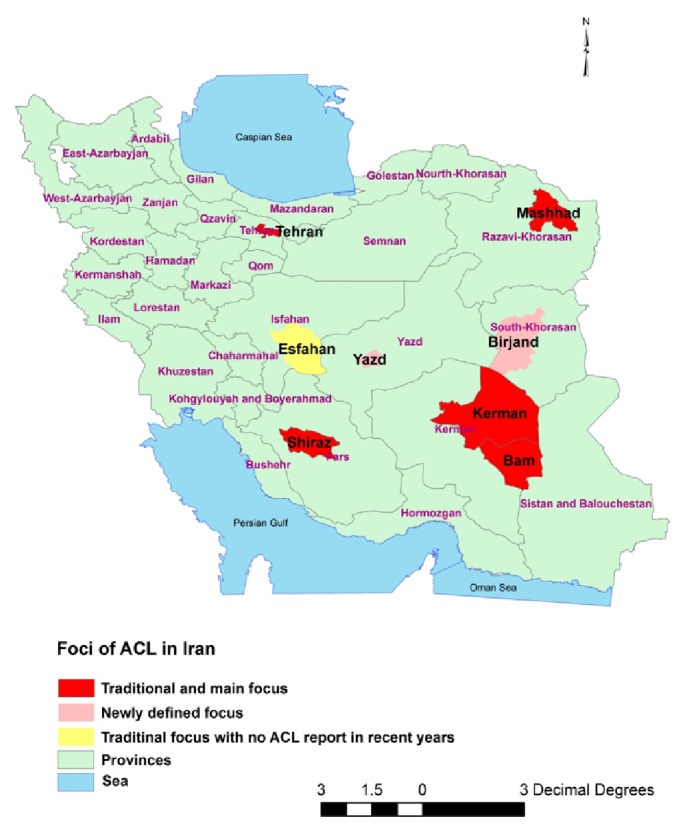
Map (down to county level) showing the main foci of* Leishmania tropica* in Iran. Kerman, Bam, Shiraz, Tehran, and Mashhad are the traditional endemic foci (red polygons). Birjand and Yazd have seen recent introductions of* L. tropica *(pink).* L. tropica* has not been reported from Esfahan in recent years (yellow).

ACL is endemic in several large cities, including Tehran, Mashhad, Yazd, Shiraz, and Kerman, and small cities and their surrounding counties such as Bam and Birjand [15–19] (Figure 1).

Population growth and displacement, urbanisation, human behaviour, anthropogenic environmental changes, drug resistance, new agricultural plans, and new parasite-vector relationship have resulted in epidemiological changes and an increased CL incidence rate in different regions [20–23]. Tracking the changing epidemiology of CL can be done with molecular taxonomy studies to determine the* Leishmania* parasite population structure and heterogeneity [24]. Different* Leishmania* genes have been used to study the phylogeny of the* Leishmania* at genus, species and strain levels, including glycoprotein 63 [25], cytochrome b [26], heat shock proteins [27], tubulin [28], N-acetylglucosamine-1-phosphate transferase [29], miniexon [30], kinetoplast minicircle DNA [31], internal transcribed spacer of ribosomal DNA (ITS-rDNA) [32], and microsatellite-containing sequences [33]. The kinetoplast DNA (kDNA) is the mitochondrial DNA of flagellated protozoa of the order kinetoplastida and consists of a network containing two types of duplex DNA circles: (i) a few dozen homogenous maxicircles (~25-50) ranging from 20 to 40 kb and (ii) ~ 5000-10,000 heterogeneous minicircle copies, 800 base pairs (bp) in size, consisting of a 600 bp variable, and a 200 bp conserved region [34, 35]. Although minicircle DNA has a critical role in the function of the trypanosomatid's mitochondrial genes by encoding guide RNAs, which edit the messenger RNA encoded by the maxicircles [36], but can be used to discriminate between strains of the same species based on the heterogeneity of its variable region [37].

Different methods in protein and DNA levels have been used for the study of* Leishmania* diversity in intraspecies level. Multilocus enzyme electrophoresis (MLEE) has been the gold standard for differentiating different species and strains of* Leishmania* based on the electrophoretic pattern of organism's enzymatic systems [38, 39]. However, MLEE is a time consuming procedure that requires the mass cultivation of parasites and has low discriminatory power at the amino acid level. It has been superceded by DNA-based techniques, including polymerase chain reaction restriction fragment length polymorphism (PCR-RFLP) of some highly variable genes, sequencing of single or multilocus genes, and multilocus microsatellite sequence typing (MLMT). MLMT and kDNA PCR-RFLP have been found to be the most powerful discriminatory approaches for intraspecies population structure studies [40–42]. There are two considerations regarding these techniques. Variations in the quantity of amplified DNA products and interlaboratory reproducibility have been reported in some studies for kDNA PCR-RFLP and MLMT is very expensive for developing countries (capillary sequencers and fluorescence labelled primers) [15].

We have already reported on the monophyletic population structure of* L. tropica* strains isolated from southeast Iran, by using sequence analysis of the rDNA-ITS locus [15, 43]. There has never been a study in Iran using kDNA PCR-RFLP to examine the intraspecies relationships in* L. tropica* from the principal ACL foci in southeast and southwest Iran. We, therefore, conducted such a study and reported our results herein.

## 2. Material and Methods

### 2.1. Study Areas and Samples

A total of 100 amastigote positive, Giemsa-stained smears were randomly collected (by using records numbers) from patients referred to the Cutaneous Leishmaniasis Control Center in Bam city and health centers in Kerman and Shiraz cities, the main foci of ACL in south Iran in 2013-14 (Figure 1). Kerman city in Kerman county is the capital of Kerman province (southeast Iran). Bam county also belongs to Kerman province and is located in the south border of Kerman county. Shiraz city is the capital of Fars province in southwest Iran. The annual incidence of CL in Kerman county was 369 cases (*L. tropica *mostly). Bam county saw 369 reported CL cases annually;* L. tropica *is reportedly the causative agent of all CL cases [18]. Shiraz city is the traditional focus of* L. tropica* in southwest Iran but recent studies show a changing epidemiology with ACL confined mostly to the central areas of the city and ZCL caused by* L. major* in the suburbs [16].

### 2.2. Microscopic Examination

Giemsa-stained slides were prepared (as per routine clinic care) from skin lesion scrapings and examined under the microscope.* Leishmania* amastigotes were sought under high power field resolution and the number of amastigotes were reported as negative (0 parasite/1000 HPF), trace (+/- or suspicious), + (1 - 10 parasites/1000 HPF), ++ (1 - 10 parasites/100 HPF), +++ (1 - 10 parasites/10 HPF), and ++++ (1 – 10 parasites/1 HPF). Only slides 2−4+ were selected for molecular evaluation.

### 2.3. DNA Extraction

Tissue scratched from the Giemsa-stained smears were collected in 1.5 ml microtubes containing lysis buffer (Tris 100 mM, EDTA 10 mM, NaCl 100 mM, SDS 1%, and Triton X100 2%). Then, 8-10 *μ*l proteinase K (10*μ*g/*μ*l) was added and the samples were vortexed and incubated at 56°C for one hour. Samples were extracted once with phenol/chloroform (25:24 v/v) and once again with chloroform. Equal volumes of isopropanol and one tenth volume of 3 M NaAc were added to precipitate the DNA. Extraction was followed by washing with 70% ethanol. The DNA precipitant was dried and then suspended in 50 *μ*l ultrapure water.

### 2.4. kDNA PCR

kDNA PCR was performed to identify the* Leishmania* species and produce enough product for the later kDNA RFLP. kDNA was amplified by using the primers 13Z (5′-ACT GGG GGT TGG GTG TAA AAT AG-3′) and LiR (5′-TCG CAG AAC GCC CCT-3′). The PCR mixture consisted of 12.5*μ*l of 2x premix (Ampliqon, Denmark), 20 pmol of each primer, 5 *μ*l of template DNA, and enough water up to 25 *μ*l. The cycling PCR conditions were 95°C for 5 min followed by 35 cycles of 94°C for 45s, 55°C for 60 s, and 72°C for 90 s and a final extension at 72°C for 7 min in a thermal cycler machine (Applied Biosystems 2700). Distilled water instead of DNA template was used in each run as a negative control. A clinical isolate with a laboratory confirmed species finding as* L. tropica* and one reference strain of* L. major *(MRHO/IR/75/ER) were used for species identification. The species specific band sizes discriminate* L. tropica* and* L. major* [37]. The PCR products were subjected to 1.2% agarose gel electrophoresis and visualized by a transilluminator. A 100 bp DNA marker was used for sizing the bands. A total of 39 smears that were identified as* L. tropica* were selected: (i) 14 from Bam district (ID sample: B18, B25 to B29, B31, B33 to B39), (ii) 12 from Kerman (B7 to B17 and B32), and (iii) 13 from Shiraz (B1 to B6, B19 to B24 and B30).

### 2.5. kDNA-Restriction Fragments Length Polymorphism

kDNA-minicircle amplicons were exposed to the restriction enzymes* Msp*I (*Hpa*II) and* Cla*I (Fermentas, Lithuania) for 2 h, in a final reaction volume of 15*μ*l containing 1 U of restriction enzyme, 1.5*μ*l of 10X buffer, 6-10 *μ*l PCR product (depending on the sharpness of the obtained band), and enough ultrapure distilled water at 37°C. An aliquot of 10-15 *μ*l of enzyme-treated products was applied in each well in the gel. The kDNA RFLP profiles were electrophoresed on 2% Metaphor agarose (Cambrex, USA) and the obtained bands were visualized by a transilluminator. A 100 bp DNA marker was used as standard to estimate the band sizes.

### 2.6. Data Analysis

The kDNA RFLP banding pattern for each isolate was analysed by NT SYS software version 2 [44]. All bands sizes were recorded in a table and scored as one for band presence and zero for band absence. Finally, the banding pattern for each isolate was recorded and two trees were inferred, using the unweighted pair group method with arithmetic mean (UPGMA), based on the banding pattern obtained using* Msp*I and* Cla*I restriction enzymes. The clusters constituting the trees were named by alphabetic letters for better interpretation. The genotypes (banding patterns) obtained using* ClaI* and* MspI* enzyme-based digestion of kDNA were named GCs and GMs, respectively, and GM-GC was used for the final genotypes from both banding patterns.

## 3. Results

**Figure 2:**
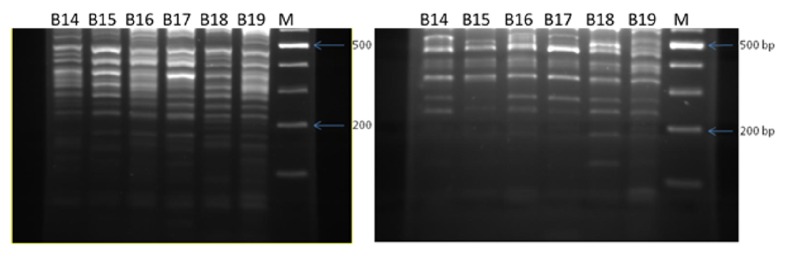
The banding patterns obtained by digestion of kDNA of the same samples by the enzymes* ClaI* (right) and* MspI* (left). The higher number of bands was obviously obtained when* MspI* was used. B14-B17 samples were from Kerman and B18 and b19 were obtained from Bam and Shiraz, respectively.

The patient's demographic and lesion data are shown in Table 1. Enzymatic digestion with both* Msp*I and* Cla*I enzymes revealed different patterns for the isolates of* L. tropica* obtained from different foci. The approximate band sizes obtained by digestion of isolates with* Msp*I were 600, 580, 550,520, 500, 480, 450, 400, 360, 330, 310, 300, 280, 270, 250, 240, 220,190, 180, and 160 bp. Likewise, different band sizes were found among PCR products digested by* ClaI*: 600, 550, 480, 450, 400, 380, 340, 280, 250, 200, and 140 bp (Figure 2).

### 3.1. Genotypes Obtained by Digestion of kDNA by ClaI

Digestion of the kDNA products of isolates obtained from Bam showed 11 genotypes among 14 isolates: GC1-GC3, GC5, GC6, GC8, GC9, GC14, GC15, GC18, and GC24. Three genotypes were found in more than one isolate: GC3 (B34 and B38), GC5 (B26 and B28), and GC9 (B18 and B39) in this focus. Among 13 isolates from Shiraz, 10 genotypes were found: GC1, GC2, GC7, and GC16-22; three isolates (B2 B4, and B6) belonged to genotype GC17 and two other isolates (B19, B21) to genotype GC19. Genotypes GC1, GC2, GC4, GC8, GC10-13, GC15, and GC23 were found from Kerman county; three (B13, B15, and B16) of 12 Kerman isolates were from one genotype (GC2).

Several genotypes were found in more than one focus: (i) two genotypes GC8 (B10-Kerman and B29-Bam) and GC15 (B17-Kerman and B31-Bam) were found in Kerman and Bam, (ii) GC18 was present in Shiraz and Bam (B5-Shiraz and B25-Bam isolates), and (iii) genotypes GC1 (B1-Shiraz, B7-Kerman and B36-Bam) and GC2 (B3-Shiraz, B33-Bam, B13-Kerman, B15-Kerman and B16-Kerman) were found in all three foci.

### 3.2. Genotypes Obtained by Digestion of kDNA with MspI

GM10, GM14, GM18, GM21-GM29, and GM34 were found among isolates obtained from Bam county. All banding patterns were different except for two isolates, B33 and B34, which were GM25. Therefore, 13 different genotypes were revealed among the 14 isolates of Bam county. The genotypes from the Kerman isolates were GM12, GM13, GM14, GM15, GM16, GM17, GM19, GM20, and GM30-GM32; two isolates (B13 and B15) belonged to the GM17 genotype, so 11 different genotypes were revealed in this focus. Only one genotype, GM14, was found in both Kerman (B9) and Bam (B18). In Shiraz, there were 11 genotypes from 13 isolates, consisting of GM1-GM9, GM11, and GM33; GM1 (B1 and B6 isolates) and GM3 (B2 and B4 isolates) were observed in two isolates.

### 3.3. Phenetic Tree Topology Based on the Banding Patterns Obtained from kDNA Digestion with ClaI Enzyme

**Figure 3:**
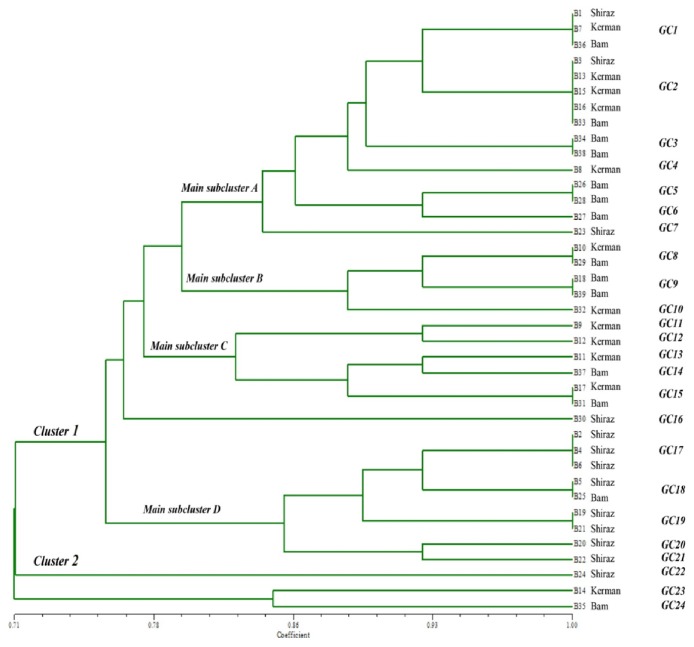
The UPGMA generated tree based on the banding patterns obtained from digestion of kDNA with* ClaI* enzyme showing clusters 1 and 2 and subclusters (A-D) of cluster 1. Subclusters A to C comprise most genotypes from Bam and Kerman and subcluster D most genotypes from Shiraz.

Two main clusters were generated on the UPMGA tree (Figure 3). Most cases were found in cluster 1, which consisted of four main subclusters A to D. Subcluster A comprised seven genotypes, GC1-GC7, from 15 isolates. GC1 and GC2 came from all three foci but GC3, GC5, and GC6 only from Bam county. GC4 and GC7 were from Kerman and Shiraz, respectively. Of the 15 isolates, 12 isolates (80%) were from Bam and Kerman. In subcluster B, three genotypes, GC8-10, were found in five isolates from Kerman and Bam counties. Subcluster C consisted of five genotypes, GC11-15, and six isolates from Kerman and Bam counties. One isolate from Shiraz (B30) was GC16 genotype that represented a separate taxon between subclusters C and D. Subcluster D consisted of five genotypes, GC17-21, from nine isolates; eight and one were from Shiraz and Bam district, respectively.

Cluster 2 was comprised of three isolates, each with a different genotype: (i) GC22 (B24) from Shiraz, (ii) GC23 (B14) from Kerman, and (iii) GC24 (B35) from Bam; the Bam and Kerman genotypes were grouped in a subcluster.

### 3.4. Phenetic Tree Topology Based on the Banding Patterns Obtained from kDNA Digestion with MspI Enzyme

**Figure 4:**
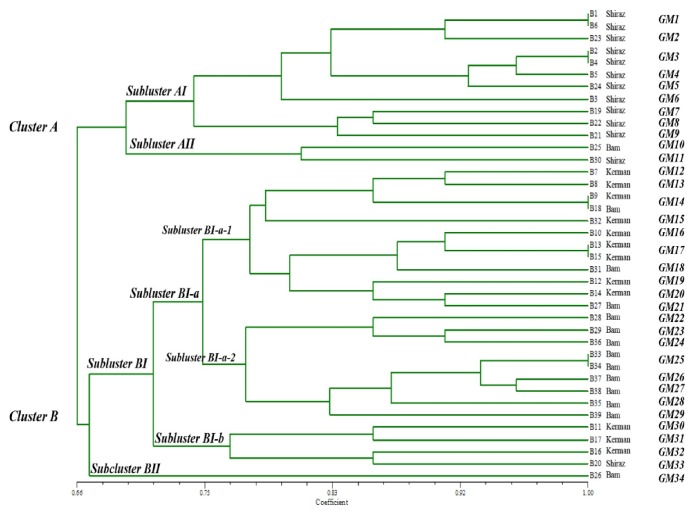
The UPGMA generated tree based on the banding patterns obtained from digestion of kDNA with* MspI* enzyme (n=34 genotypes). Cluster A isolates, except one, were from Shiraz. Cluster B included only isolates from Kerman and Bam.

In this phenetic tree, there were two obvious clusters (A and B) for the* L. tropica *strains (Figure 4**)**. Cluster A contained 13 isolates; 12 from Shiraz and only one from Bam and had two main subclusters, AI and AII. The AI subcluster contained nine genotypes, including GM1-GM9 genotypes, all from Shiraz. Subcluster AII comprised genotypes GM10 and GM11 from Bam and Shiraz, respectively.

Cluster B subclustered into BI and BII subclusters, containing 25 and one isolate, respectively, with further division of the BI subcluster into subclusters BI-a and BI-b and, in turn, branching of the BI-a into two main groups, BI-a-1 and BI-a-2. The BI-a-1 group contained 10 genotypes, GM12-21 (12 isolates) from Bam and Kerman, whereas the BI-a-2 group contained eight genotypes (GM22-29) only from Bam county. Subcluster BI-b contained four genotypes, GM30-33 (4 isolates), three from Kerman and one from Shiraz county. One isolate from Bam comprised subcluster BII.

Although 24 and 34 banding patterns were obtained from the digestion of kDNA with* Cla*I and* Msp*I enzymes, respectively, isolates B13 and B15 (Kerman) and B2 and B4 (Shiraz) showed the same genotypes in both enzymatic digestions that were identified GM17-GC2 and GM3-GC17. Therefore, a total of 37 different genotypes were identified from 39* L. tropica* isolates from southern Iran based on the both banding patterns obtained from the digestion by both enzymes for each sample (Table 2).

## 4. Discussion

In this study, a total of 37 genotypes were identified from 39* L. tropica* isolates obtained from three main foci of ACL in southern Iran, Shiraz (southwest) and Bam and Kerman (southeast). The UPGMA tree topology, based on* Msp*I enzyme digested kDNA, showed well-differentiated* L. tropica* strains in two main clusters with a predominance of Shiraz in Cluster A and B group subclustered into mixed Kerman Bam isolates or Bam isolates alone. The tree topology obtained from* Cla*I digested kDNA distinguished southwest from southeast isolates but was unable discriminate between two main foci of southeast Iran. The* Cla*I enzyme has fewer cutting sites in the kDNA and has intrinsically less discriminating power than the* Msp*I enzyme. Nevertheless, both enzymes had broadly consistent results.

There are distinct geographical, environmental, and climatic differences between southeast and southwest Iran. The southeast has a predominantly desert climatic with high temperatures, low rainfall, and low flora and fauna diversity and density; the southwest is a mountainous region that sees greater rainfall and is covered with more green land cover, including pastures and forests [45, 46]. These differences may have led to the evolution of different* L. tropica* populations.

We have already shown that a monophyletic structure of* L. tropica* exists in southeast Iran based on the sequence analysis of ITS-rDNA [15] and that there is close genetic relationship between east and southeast Iranian* L. tropica* [17]. Moreover, two populations of* L. tropica* are present in Herat, west Afghanistan, in which the minor population was found to be more homogenous with southeast Iranian isolates and was assumed to have originated from that population [47]. ITS-rDNA analysis of Shiraz* L. tropica* isolates also distinguished two subgroups; one group was clustered with southeast and east* L. tropica* and another group was distributed in the another main cluster and was found to be more similar to northeast isolates [17]. Herat region in Western Afghanistan and Shiraz city in southwest Iran are mountainous regions in contrast to arid desert regions which surround the southeast-east regions. We hypothesize that climate affects the sandfly life cycle and evolution and that aridity and humidity are important drivers for* L. tropica* diversity in Iran which we believe it explains the* L. tropica* genetic diversity between humid western Afghanistan and arid east-southeast Iran [48]. Aridity along with some other human and climatic factors also affected the distribution of visceral leishmaniasis in southwest Iran as one of the two main foci of VL in Iran where aridity confined the distribution of VL [48–50].

Sequence variation among the kinetoplastid minicircle of* Leishmania* species occurs rapidly in nature [34]. Therefore, in comparison to ITS sequence analysis, kDNA PCR-RFLP is able to reveal more difference between* L. tropica* populations. This explains the diversity and subclustering seen in some* L. tropica* isolates from southeast foci and the divergence between Bam and Kerman* L. tropica* populations in comparison to previous data obtained by ITS sequence analysis in south Iran [15]. Bam and Kerman are adjoining counties in Kerman province that are surrounded by large deserts and the mean annual rainfall showed arid weather in both foci but there are some environmental differences between them. The presence of hundreds hectares of orchards, including palm and citrus trees, in Bam and its rural environs can be seen as green images by satellite data analysis [51]. These vegetation and crop residue and the local humidity resulting from irrigation produce suitable breeding microfoci for* P. sergenti*, the main vector for transmitting* L. tropica* in Iran. In addition, many agricultural workers are exposed to these sandflies while they work in the orchards and this probably explains the fivefold higher CL incidence in Bam county compared to Kerman despite having a lower population. 71% and 14% of all CL cases have occurred in Bam and Kerman counties in Kerman province [18]. The Bam Kerman environmental differences may also have affected the* P. sergenti* strains and help explain the genetic variation in* L. tropica* strains from these foci of southeast Iran.

Previously, there had been movement of people between Bam and Kerman but following the devastating earthquake in Bam in 2003 and massive destruction of buildings (more than 70%), many people fled from Bam to live in neighbouring cities and villages, particularly Kerman city [52, 53]. The cluster B group analysis found that the subcluster B1-a-2 comprised isolates from Bam county (which included most isolates from Bam county in the tree) but subcluster B1-a-1 consisted of isolates from both Kerman and Bam. This topology supports the notion of an influx of* L. tropica* strains and gene flow by the immigration of people from Bam to Kerman. Other studies have showed heterogeneity of* Leishmania* in limited geographical areas using kDNA as a high-resolution marker. Using a minisatellite probe and kDNA RFLP analysis for genomic fingerprinting, two clusters of* L. tropica* strains were found from Kfar Adunim village in the Judean desert in the Palestinian West Bank. One strain was inconsistent with other strains from the area, suggesting it was imported from another endemic focus [54]. Another study showed that a man made barrier wall has decreased human and Canidae movements (Canidae are reservoirs of* L. infantum*) and resulted in the divergence of two separate clusters of* L. infantum *isolates in two geographical regions either side of the wall [31].

Salvatore* et al*. (2016) studied the heterogeneity of* L. infantum* isolates obtained from dogs in seven regions in Northern Italy and showed five different kDNA RFLP patterns that were specific to geographical origin [55]. Similar findings have been reported from Portugal. Of 13* L. infantum* genotypes obtained among 120 isolates by kDNA RFLP-RFLP, only three were found country wide while the other 10 genotypes could discriminate* L. infantum* population in northern focus from those in central and southern regions [56]. In Majorca island,* L. infantum* has less variability compared to isolates from mainland Spain and can be explained by its geographical isolation from the mainland resulting in an ecological niche with limited canine and human movement into Majorca [57].

Environmental differences between geographical regions may directly affect vector populations and their parasites due to probable parasite-vector coevolution. Kuhls* et al*. (2008) showed that sandfly vectors might play a role in sustaining genetic diversity of* Leishmania* [58] and other studies suggest sandfly vectors might play a role in selecting specific parasite strains at a regional level and, therefore, contribute to the genetic structure of* Leishmania* [59, 60]. The existence of two genetically divergent populations of* L. tropica* in southeast and southwest Iran may be due to specific strains of* P. sergenti* sandflies that have adapted to the specific climatic and environmental conditions in these two regions. Based on sequence analyses of mitochondrial genes, Moin-Vaziri* et al*. (2007) demonstrated three lineages of* P. sergenti* strains in Iran, which diverged according to geographical region into southwest, northwest-central-southeast, and northeast Iran [61]. Although these findings are consistent with divergence of Iranian southeast and southwest* L*.* tropica, *they are discordant with our* L. tropica* genetic data that show a divergence of the southeast* L. tropica* genotypes [61].

Compared to other countries and* Leishmania* species studies, the highest number of different* L. tropica* genotypes is found in southern Iran based on kDNA RFLP patterns [31, 34, 40, 55–57, 59, 60, 62] and the current study has confirmed higher* L. tropica* heterogeneity which previously was found in comparison to other species [42]. The number of genotypes obtained with kDNA PCR-RFLP depends on the* Leishmania* species, the type of digesting enzyme, and cutting sites. To the best of our knowledge, the only other* L. tropica* study that used kDNA PCR-RFLP was a study from northern Palestine; in that study, the genetic heterogeneity was less compared to our study [60]. Clonal propagation of an asexually reproducing organism is the main method for the proliferation of* Leishmania *but sexual recombination is also considered important for producing genetic diversity as evidenced by the identification of hybrid species like* L. major* and* L. arabica* in Saudi Arabia and* L. braziliensis* and* L. panamensis* in Nicaragua [63–65] and studies reporting evidences of recombination in* Leishmania *species in the New and Old Worlds [41, 66–70]. Ghatee* et al*. (2013) showed at least two different alleles with a 100 bp gap in one allele for ITS-rDNA in Iranian* L. tropica* isolates and proposed that sexual recombination caused this 100 bp DNA fragment deletion/insertion; this may also partly explain the considerable genetic variations found in our current study [43]. Evidences of existence of at least two alleles for ITS locus of* L. tropica* from some other endemic foci in the World have been reported by Schönian* et al*. (2001) and Mauricio* et al*. (2004) [42, 71].

## 5. Conclusion

Using kDNA PCR-RFLP we found considerable heterogeneity in* L. tropica* from southern Iran, 37 genotypes from 39 isolates, which may be part explained by sexual recombination that previously was hypothesized for Iranian* L. tropica*. A clear distinction between southeast and southwest and divergence with overlapping in the former were found in* L. tropica* populations in south Iran that may have been driven by the different geographical and climatic conditions between these regions. Increased human movement after the 2003 earthquake in Bam probably resulted in mixing of* L. tropica* strains in the southeastern foci. These results will be used for recognition and following up epidemiological changes as well as for control measures and also for further kDNA RFLP studies in other ACL foci in northeast and central regions of Iran to complete the* L. tropica* genetic picture in Iran.

## Figures and Tables

**Table 1:** The patient's demographic and lesion data.

Patient's data	percent
Sex	
Male	48.7
Female	51.3
Total	100
Age	
<10	28.2
10-20	23.1
20-60	41
>60	7.7
Total	100
Lesion sites	
Face and neck	35.9
One hand	38.4
Both hands	7.7
One leg	12.8
Both legs	2.6
Hand and leg	2.6
Total	100

**Table 2:** The samples ID, geographical origin, and related GM, GC, and GM-GC genotypes (banding patterns).

Isolate ID	Geographical origin			Genotypes		
	Bam (SE)	Kerman (SE)	Shiraz (SW)	GM	GC	GM-GC
B1			✔	GM1 ^*∗*^	GC1 ^*∗∗∗*^	GM1-GC1
B2			✔	GM3 ^*∗*^	GC17 ^*∗*^	GM3-GC17
B3			✔	GM6	GC2 ^*∗∗∗*^	GM6-GC2
B4			✔	GM3 ^*∗*^	GC17 ^*∗*^	GM3-GC17
B5			✔	GM4	GC18 ^*∗∗*^	GM4-GC18
B6			✔	GM1 ^*∗*^	GC17 ^*∗*^	GM1-GC17
B7		✔		GM12	GC1 ^*∗∗∗*^	GM12-GC1
B8		✔		GM13	GC4	GM13-GC4
B9		✔		GM14	GC11	GM14-GC11
B10		✔		GM16	GC8 ^*∗∗*^	GM16-GC8
B11		✔		GM30	GC13	GM30-GC13
B12		✔		GM19	GC12	GM19-GC12
B13		✔		GM17 ^*∗*^	GC2 ^*∗∗∗*^	GM17-GC2
B14		✔		GM20	GC23	GM20-GC23
B15		✔		GM17 ^*∗*^	GC2 ^*∗∗∗*^	GM17-GC2
B16		✔		GM32	GC2 ^*∗∗∗*^	GM32-GC2
B17		✔		GM31	GC15 ^*∗∗*^	GM31-GC15
B18	✔			GM14	GC9 ^*∗*^	GM14-GC9
B19			✔	GM7	GC19 ^*∗*^	GM7-GC19
B20			✔	GM33	GC20	GM33-GC20
B21			✔	GM9	GC19 ^*∗*^	GM9-GC19
B22			✔	GM8	GC21	GM8-GC21
B23			✔	GM2	GC7	GM2-GC7
B24			✔	GM5	GC22	GM5-GC22
B25	✔			GM10	GC18 ^*∗∗*^	GM10-GC18
B26	✔			GM34	GC5 ^*∗*^	GM34-GC5
B27	✔			GM21	GC6	GM21-GC6
B28	✔			GM22	GC5 ^*∗*^	GM22-GC5
B29	✔			GM23	GC8 ^*∗∗*^	GM23-GC8
B30			✔	GM11	GC16	GM11-GC16
B31	✔			GM18	GC15 ^*∗∗*^	GM18-GC15
B32		✔		GM15	GC10	GM15-GC10
B33	✔			GM25 ^*∗*^	GC2 ^*∗∗∗*^	GM25-GC2
B34	✔			GM25 ^*∗*^	GC3 ^*∗*^	GM25-GC3
B35	✔			GM28	GC24	GM28-GC24
B36	✔			GM24	GC1 ^*∗∗∗*^	GM24-GC1
B37	✔			GM26	GC14	GM26-GC14
B38	✔			GM27	GC3 ^*∗*^	GM27-GC3
B39	✔			GM29	GC9 ^*∗*^	GM29-GC9

*∗* indicates that the genotypes were found in more than one isolate in one geographical focus.

*∗∗* and *∗∗∗* indicate that the genotypes were found in more than one isolate in two and all three geographical foci, respectively.

The underlined GM-GC genotypes (GM17-GC2 and GM17-GC2) are found in more than one isolate. 37 GM-GC genotypes were identified from 39 L. tropica isolates.

## Data Availability

Access to these data will be considered by the author upon request.
